# The Prevalence of Primary Pediatric Prehypertension and Hypertension in a Real-World Managed Care System

**DOI:** 10.1111/jch.12173

**Published:** 2013-08-07

**Authors:** Corinna Koebnick, Mary H Black, Jun Wu, Mayra P Martinez, Ning Smith, Beatriz D Kuizon, Steven J Jacobsen, Kristi Reynolds

**Affiliations:** 1 Department of Research and Evaluation, Kaiser Permanente Southern CaliforniaPasadena, CA; 2 Center for Health Research, Kaiser Permanente NorthwestPortland, OR; 3 Pediatric Nephrology Department of Pediatrics, Kaiser Permanente Los Angeles Medical CenterLos Angeles, CA

## Abstract

To assess the burden associated with hypertension, reliable estimates for the prevalence of pediatric hypertension are vital. For this cross-sectional study of 237,248 youths aged 6 to 17 years without indication of secondary hypertension, blood pressure (BP) was classified according to age, sex, and height using standards from the Fourth Report on the Diagnosis, Evaluation, and Treatment of High Blood Pressure in Children and Adolescents as prehypertension with at least 1 BP ≥90th percentile and as hypertension with 3 BPs ≥95th percentile. The prevalence of prehypertension and hypertension were 31.4% and 2.1%, respectively. An additional 21.4% had either 1 (16.6%) or 2 (4.8%) BPs ≥95th percentile. Based on this large population-based study using routinely measured BP from clinical care, a remarkable proportion of youth (6.9%) has hypertension or nearly meets the definition of hypertension with 2 documented BPs in the hypertensive range.

Hypertension is one of the leading causes of global disease burden.^1^–^4^ Early diagnosis, evaluation, and treatment of hypertension are important to alleviate the health risks associated with hypertension.^5^,^6^ End-organ damage in the form of cardiac structural changes, a consequence of hypertension, can be present in adolescent and early adult life.^8^–^9^

In contrast to hypertension secondary to causes such as renal disease, primary hypertension is usually asymptomatic and often remains undiagnosed.^10^,^11^ To assess future economic and health care demands resulting from the significant disease burden associated with hypertension, reliable estimates for the prevalence of pediatric hypertension are vital. Recent studies suggest that the prevalence of hypertension in youth^13^–^14^ and young adults^15^ is increasing. According to previous studies, the prevalence of hypertension in youth, identified by ≥3 blood pressures (BPs) ≥95th percentile, is between 0.1% and 5%.^11^,^16^ However, previous studies in youth are inconclusive because of small sample size,^11^ limited follow-up to confirm hypertension,^16^ and differences in clinical^11^–^16^ vs school-based settings.^17^

In the present population-based, cross-sectional study, we provide estimates of the prevalence of prehypertension and hypertension in youth from routine clinical care in an integrated care organization. We also provide detailed information on disparities in hypertension prevalence across different racial/ethnic groups and on the prevalence of prehypertension and hypertension according to the frequency of elevated BP measures.

## Patients and Methods

### Study Design and Patients

Patients enrolled in this study were pediatric members of a prepaid integrated health plan between January 1, 2007, and December 31, 2009. Kaiser Permanente Southern California (KPSC) is the largest health care provider in Southern California. In 2012, KPSC provided health care services to more than 3.6 million members, approximately 22% of whom were 17 years or younger.^18^ Detailed demographic characteristics of the KPSC membership population are described elsewhere.^18^ Members receive care in medical offices and hospitals managed by KPSC. A comprehensive electronic health record (EHR) system, HealthConnect (Kaiser Permanente, Oakland, CA), was implemented region-wide prior to 2007. The study protocol was reviewed and approved by the institutional review board of KPSC, which granted a waiver for the requirement of an informed consent.

**Figure 1:**
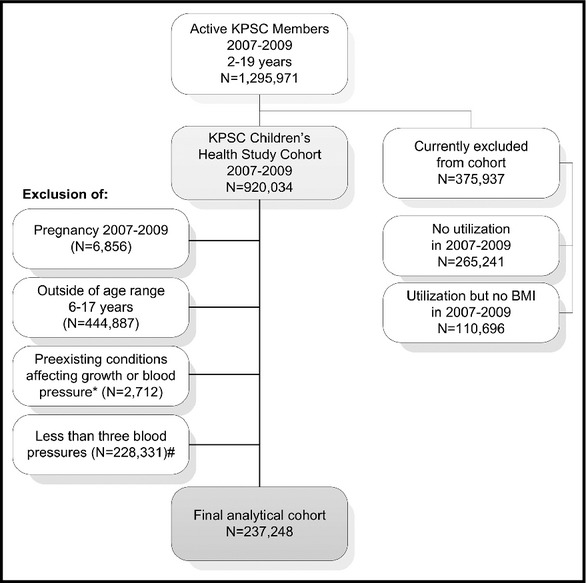
Flow chart of the Kaiser Permanente Southern California (KPSC) Children's Health Study and further inclusions for the final analytical cohort in the present study. *Existing diagnoses of chronic conditions significantly affecting growth or blood pressure (n=2712), such as growth hormone deficiency (*International Classification of Disease, Ninth Revision* [*ICD-9*] 253.3) or overproduction (*ICD-9* 253.0), aortic coarctation (*ICD-9* 747.10), chronic renal disease (*ICD-9* 585.x), congenital adrenal hyperplasia (*ICD-9* 255.2), Cushing syndrome (*ICD-9* 255.0), hyperaldosteronism (*ICD-9* 255.1), and/or hyperthyroidism (*ICD-9* 242). #Except youth patients with a diagnosis of essential hypertension (*ICD-9* 401 or 402) and at least one prescription of antihypertensive drugs (n=984) were classified as patients with hypertension if there was no information in the electronic health record to suggest a different diagnosis.

For this cross-sectional study, we used EHR data from a subset of patients enrolled in a large population-based cohort, the KPSC Children's Health Study, from January 1, 2007, through December 31, 2009.^19^ The date of the first available BP was considered the date of study enrollment. As shown in Figure 1, we excluded patients who were younger than 6 years or older than 17 years (n=444,887) and patients who became pregnant anytime during the 36-month study period (n=6856). We also excluded patients with ≥1 pre-existing diagnoses of chronic conditions known to significantly affect growth or BP (n=2712), such as growth hormone deficiency (*International Classification of Disease, Ninth Revision* [*ICD-9*] 253.3) or overproduction (*ICD-9* 253.0), aortic coarctation (*ICD-9* 747.10), chronic renal disease (ICD-9 585.x), congenital adrenal hyperplasia (*ICD-9* 255.2), Cushing syndrome (*ICD-9* 255.0), hyperaldosteronism (*ICD-9* 255.1), and/or hyperthyroidism (*ICD-9* 242). Patients who had filled a prescription for an antihypertensive medication and who had at least 1 outpatient diagnosis of hypertension (*ICD-9* 401, 402, 403, or 404) (n=984) prior to study enrollment were identified as having hypertension. Among the remaining youth, BP measurements at 3 of 4 consecutive, separate clinic visits during 36 months were required for the identification of hypertension.^20^ We therefore excluded participants of the KPSC Children's Health Study with fewer than 3 BP measurements within 36 months following the date of study enrollment (n=228,331). This resulted in a final analytical cohort of 237,248 children and adolescents aged 6 to 17 years.

### BP Measurements and Classification

BP was measured routinely at almost every outpatient visit. Nurses, medical assistants, and physicians were trained according to guidelines of the American Association of Critical Care Nurses for pediatric care.^21^ Digital devices (Welch Allyn Connex series; Welch Allyn Inc, Skaneateles Falls, NY) are the preferred BP measurement devices at KPSC. In some cases, a wall-mounted aneroid sphygmomanometer (Welch Allyn Inc) was used. The cuff size was estimated after inspection of the bare upper arm at the midpoint between the shoulder and elbow using a bladder width approximately 40% of the arm circumference. Personnel were also trained to ensure that the bladder inside the cuff encircled 80% to 100% of the circumference of the arm in children younger than 13 years according to standard recommendations. A full range of different cuff sizes was available at the BP reading station. BPs were measured in children while in a seated position after at least 3 to 5 minutes of rest with the midpoint of the arm supported at heart level. The brachial artery was palpated, the cuff was placed to ensure that that midline of the bladder was over the arterial pulsation, and then the cuff was snugly wrapped and secured around the child's bare upper arm. In the pediatric setting, nurses and medical assistants are instructed to repeat readings for elevated BP. If the mean BP is elevated, the primary care provider will measure BP using an auscultatory device in the examination room. However, repeated readings are not systematically recorded in the electronic medical record and from electronic data and aneroid readings cannot be distinguished from oscillometric readings. All personnel measuring BP are certified in BP measurement at the time of hiring and recertified annually. In pediatric care, staff must complete a Web-based training session and successfully pass a certification process that includes knowledge of preparing patients for measuring BP, selecting correct cuff size, and using standard techniques for BP measurement. Additionally, staff must demonstrate competency in measuring BP through direct observation. However, intensity of in-house training may vary by medical center, and deviations of the preferred measurement method may have occurred.

BP measures during 36 months following the date of study enrollment were extracted from the EHR for all outpatient encounters in which the presence of fever was not indicated (body temperature >100.4°F or >38.0°C). The rationale for the 36-month study period was to allow the use of 3 regular annual visits to be included in the classification of BP. A lack of follow-up visits scheduled may lead to an underestimation of the prevalence of hypertension. Because of the 36-month study period, we used the first 4 consecutive BPs, thereby allowing 1 BP to be outside of the requirements. We classified BP using the recommendations of the Fourth Report On the Diagnosis, Evaluation, and Treatment of High Blood Pressure in Children and Adolescents of the National High Blood Pressure Education Program (NHBPEP)^20^ combined with the recommendations for adults of the Seventh Report of the Joint National Committee on Prevention, Detection, Evaluation, and Treatment of High Blood Pressure (JNC 7).^22^ Prehypertension was defined as at least 1 BP between the 90th percentile and <95th percentile (or ≥120/80 mm Hg even if lower than the 90th percentile). Because of high variability of BP in this population, the NHBPEP definition of hypertension in children and adolescents requires a BP ≥95th percentile (or ≥140/90 mm Hg even if lower than the 95th percentile) on at least 3 separate occasions. We classified youth with 1 or 2 BPs ≥95th percentile as “blood pressure in the hypertensive range.” As described above, patients with a diagnosis of essential hypertension (*ICD-9* 401 or 402) and at least one prescription of antihypertensive drugs (n=984) were classified as patients with hypertension if there was no information in the EHR to suggest a different diagnosis.

### Race and Ethnicity

We obtained race and ethnicity information from health plan administrative records and birth records. We categorized race/ethnicity as Hispanic (regardless of race), non-Hispanic white, black, Asian or Pacific Islander, and other or unknown race/ethnicity. A validation study comparing health plan administrative records and birth certificate records of 325,810 children was described in detail elsewhere.^23^ When race and ethnicity information was unknown (31.7%), administrative records were supplemented by an imputation algorithm based on surname lists and address information derived from the US Bureau of Census.^24^,^25^ The specificity and positive predictive values for the imputation were >98% for all races/ethnicities.^27^

### Body Weight and Height

Body weight and height are routinely measured and were extracted from electronic health records. Body mass index (BMI) was calculated as weight (kilograms) divided by the square of the height (meters). Definitions of overweight and obesity in children and adolescents are based on the sex-specific BMI-for-age growth charts developed by the Centers for Disease Control and Prevention and the World Health Organization definitions for overweight and obesity in adults.^28^,^29^ Children were categorized as underweight (BMI for age <5th percentile), normal weight (BMI for age ≥5th to <85th percentile), overweight (BMI for age ≥85th to <95th percentile or a BMI ≥25 to <30 kg/m^2^), moderately obese (BMI for age ≥95th to <1.2 × 95th percentile or a BMI ≥30 to <35 kg/m^2^), and extremely obese (BMI for age ≥1.2 × 95th percentile or a BMI ≥35 kg/m^2^).

### Socioeconomic Status

To define socioeconomic status, we used neighborhood education estimated based on the linkage of health plan members addresses via geocoding with US census block data.^31^

### Statistical Analysis

Differences in the distribution of basic demographics for the analytical cohort, as well as patients excluded due to BP measurement requirements, were assessed using the chi-square test. For each patient, age was assessed on the day the first BP was measured. The prevalence of prehypertension and hypertension was estimated for the entire cohort and by sex (boys, girls), age group (6–11 years, 12–17 years), race (Non-Hispanic white, Hispanic, black, Asian or Pacific Islander, other or unknown), and state-subsidized insurance (Yes/No). The prevalence was expressed as a percentage with corresponding 95% confidence intervals (CIs). We examined the associations of prehypertension and hypertension with age, sex, race, and weight class by using log-binomial regression models to estimate the crude prevalence ratio (PR) and corresponding 95% CIs. A multivariable model was used to adjust for age, sex, race, and weight class. In order to detect the possible interactions of age by race and sex by race on prehypertension and hypertension, we used 2 log-binomial regression models: (1) the multivariable model stratified by age or sex, and (2) additionally including 2-way interaction terms into the model. All analyses were conducted using SAS 9.2 (SAS Institute Inc, Cary, NC) (Table 1).

**Table I:** Classification of BP According to NHBPEP^20^ Combined With the Recommendations for Adults According to JNC 7^22^

Blood Pressure Classification	Systolic or Diastolic BP
Normal BP	<90th percentile
Prehypertension	≥90th percentile and <95th percentile (or ≥120/80 mm Hg even if lower than the 90th percentile)
BP in the hypertensive range (but no hypertension)^a^	≥95th percentile at ≥1 but <3 occasions (or ≥140/90 mm Hg even if lower than the 90th percentile)
Hypertension	≥95th percentile elevated on ≥3 occasions (or ≥140/90 mm Hg even if lower than the 90th percentile)

Abbreviations: NHBPEP, the Fourth Report on the Diagnosis, Evaluation, and Treatment of High Blood Pressure in Children and Adolescents of the National High Blood Pressure Education Program; JNC 7, Seventh Report of the Joint National Committee on Prevention, Detection, Evaluation, and Treatment of High Blood Pressure.

a Because classification of hypertension requires persistently elevated blood pressure (BP) with 3 BPs ≥95th percentile, an additional group was created to represent children with 1 or 2 BPs ≥95th percentile.

## Results

The study sample was comprised of 237,248 youth, of whom approximately half were Hispanic (Table 2). Compared with youth excluded from the analysis because they did not have 3 independent BPs in the study period (n=228,331), the study cohort was similar in the distribution of sex, race/ethnicity, neighborhood education, and neighborhood income. However, youth excluded from the analysis were slightly younger, and, for a significant proportion of these youth, (37.4%) the membership with medical care coverage at KPSC ended before the end of the study period.

**Table II:** Demographic Characteristics of the Study Population Compared With Patients Excluded From the Study

	Study Population			
	Included		Excluded^a^	
	No.	%	No.	%
No.	237,248	51.0	228,331	49.0
Male	115,991	48.9	118,908	52.1
Age group, y				
6–11	98,175	41.4	108,288	47.4
12–17	139,073	58.6	120,043	52.6
Race/ethnicity				
Non-Hispanic white	57,849	24.4	44,580	19.5
Hispanic	119,667	50.4	117,353	51.4
Non-Hispanic Black	16,044	6.8	16,192	7.1
Asian or Pacific Islander	14,582	6.1	15,070	6.6
Other/unknown	29,106	12.3	35,136	15.4
Neighborhood education				
Less than high school	64,798	27.3	66,211	29.0
High school graduate	50,552	21.3	49,303	21.6
Some college or associate degree	72,737	30.7	68,852	30.2
Bachelor degree or higher	49,160	20.7	43,963	19.3
Neighborhood income, $				
<15,000	22,720	9.6	23,997	10.5
15,000–34,999	42,688	18.0	43,899	19.2
35,000–49,999	32,848	13.8	32,731	14.3
50,000–74,999	46,170	19.5	44,577	19.5
75,000–99,000	33,617	14.2	31,330	13.7
100,000–149,999	36,465	15.4	32,561	14.3
≥150,000	22,740	9.6	19,236	8.4
State subsidized care^b^	39,708	16.7	26,926	11.8
Membership^c^ ended	31,742	13.4	85,500	37.4

a Due to the requirement of 3 independent blood pressures for the diagnosis of hypertension, we excluded participants of the Kaiser Permanente Southern California Children's Health Study with fewer than 3 blood pressure measurements after study enrollment except those who were taking antihypertensive drugs and had at least one outpatient diagnosis of hypertension (*International Classification of Disease, Ninth Revision* 401, 402, 403, or 404).

b Beneficiary of Medi-Cal or other state subsidized support programs.

c Membership with coverage of medical care.

The prevalence of prehypertension and hypertension were 31.4% and 2.1%, respectively (Table 3). However, a significant proportion of youth had 1 (16.6%) or 2 (4.8%) BPs in the hypertensive range (Table 3). Until the end of the 36-month study period, 10.1% (n=3990) of youth with 1 BP in the hypertensive range and 48.1% of children (n=5465) with 2 BPs in the hypertensive range had ≥3 BPs ≥95th percentile. This translates into 6.8% of youth with ≥3 BPs ≥95th percentile at the end of the 36-month study period in the entire cohort—including 2.1% with hypertension defined based on the first 4 BPs available.

**Table III:** Prehypertension and Hypertension Among Youth Aged 6 to 17 Years in Southern California Based on 4 Consecutive BPs

	BP in the Hypertensive Range							
	Prehypertension		Hypertension		One BP ≥95th Percentile^a^		Two BP ≥95th Percentile	
Variable	No.	% (95% CI)	No.	% (95% CI)	No.	% (95% CI)	No.	% (95% CI)
Total	74,501	31.4 (31.2–31.6)	39,502	16.6 (16.5–16.8)	11,373	4.8 (4.7–4.9)	5039	2.1 (2.1–2.2)
Sex								
Male	42,981	37.1 (36.8–37.3)	20,242	17.5 (17.2–17.7)	5894	5.1 (5.0–5.2)	2653	2.3 (2.2–2.4)
Female	31,520	26.0 (25.8–26.4)	19,260	15.9 (15.7–16.1)	5479	4.5 (4.4–4.6)	2386	2.0 (1.9–2.1)
Age group, y								
6–11	19,783	20.2 (19.9–20.4)	18,221	18.6 (18.3–18.8)	5280	5.4 (5.2–5.5)	2127	2.2 (2.1–2.3)
12–17	54,718	39.3 (30.1–39.6)	21,281	15.3 (15.1–15.5)	6093	4.4 (4.3–4.5)	2912	2.1 (2.0–2.2)
Race/ethnicity								
Non-Hispanic white	20,166	34.9 (34.5–35.3)	9106	15.7 (15.4–16.0)	2405	4.2 (4.0–4.3)	1024	1.8 (1.7–1.9)
Hispanic	35,443	29.6 (29.4–29.9)	20,467	17.1 (16.9–17.3)	6078	5.1 (5.0–5.2)	2814	2.4 (2.3–2.4)
Black	5240	32.7 (31.9–33.4)	2730	17.0 (16.4–17.6)	751	4.7 (4.4–5.0)	314	2.0 (1.7–2.2)
Asian or Pacific Islander	4169	28.6 (27.9–29.3)	2454	16.8 (16.2–17.4)	729	5.0 (4.7–5.4)	347	2.4 (2.1–2.6)
Other/unknown	9483	32.6 (32.0–33.1)	4745	16.3 (15.9–16.7)	1410	4.8 (4.6–5.1)	540	1.9 (1.7–2.0)
State subsidized care^a^								
No	63,272	32.0 (31.8–32.2)	32,575	16.5 (16.3–16.7)	9295	4.7 (4.6–4.8)	4086	2.1 (2.0–2.1)
Yes	11,229	28.3 (27.8–28.7)	6927	17.4 (17.1–17.8)	2078	5.2 (5.0–5.5)	953	2.4 (2.3–2.6)
Boys								
Age group, y								
6–11	10,747	21.0 (20.6–21.3)	9497	18.5 (18.2–18.9)	2704	5.3 (5.1–5.5)	1098	2.1 (2.2–2.3)
12–17	32,234	49.8 (49.4–50.2)	10,745	16.6 (16.3–16.9)	3190	4.9 (4.8–5.1)	1555	2.4 (2.3–2.5)
Race/ethnicity								
Non-Hispanic white	11,604	41.1 (40.5–41.7)	4441	15.7 (15.3–16.2)	1195	4.2 (4.0–4.5)	495	1.8 (1.6–1.9)
Hispanic	20,521	35.1 (34.8–35.5)	10,712	18.3 (18.0–18.7)	3241	5.6 (5.4–5.7)	1558	2.7 (2.5–2.8)
Black	2895	37.4 (36.3–38.5)	1295	16.7 (15.9–17.6)	332	4.3 (3.8–4.7)	132	1.7 (1.4–2.0)
Asian or Pacific Islander	2645	34.4 (33.3–35.4)	1433	18.6 (17.8–19.5)	448	5.8 (5.3–6.3)	220	2.9 (2.5–3.2)
Other/unknown	5316	38.2 (37.4–39.0)	2361	17.0 (16.3–17.6)	678	4.9 (4.5–5.2)	248	1.8 (1.6–2.0)
State subsidized care^a^								
No	36,452	37.9 (37.6–38.2)	16,642	17.3 (17.1–17.5)	4789	5.0 (4.8–5.1)	2128	2.2 (2.1–2.3)
Yes	6529	33.0 (32.3–33.6)	3600	18.2 (17.6–18.7)	1105	5.6 (5.3–5.9)	525	2.7 (2.4–2.9)
Girls								
Age group, y								
6–11	9036	19.3 (18.9–19.6)	8724	18.6 (18.2–18.9)	2576	5.5 (5.3–5.7)	1029	2.2 (2.1–2.3)
12–17	22,484	30.3 (29.9–30.6)	10,536	14.2 (13.9–14.4)	2903	3.9 (3.8–4.1)	1357	1.8 (1.7–1.9)
Race/ethnicity								
Non-Hispanic white	8562	28.9 (28.4–29.4)	4665	15.8 (15.3–16.2)	1210	4.1 (3.9–4.3)	529	1.8 (1.6–1.9)
Hispanic	14,922	24.4 (24.0–24.7)	9755	15.9 (15.6–16.2)	2837	4.6 (4.5–4.8)	1256	2.1 (1.9–2.2)
Black	2345	28.3 (27.3–29.2)	1435	17.3 (16.5–18.1)	419	5.1 (4.6–5.5)	182	2.2 (1.9–2.5)
Asian or Pacific Islander	1524	22.1 (21.2–23.1)	1021	14.8 (14.0–15.7)	281	4.1 (3.6–4.6)	127	1.8 (1.5–2.2)
Other/unknown	4167	27.4 (26.7–28.2)	2384	15.7 (15.1–16.3)	732	4.8 (4.5–5.2)	292	1.9 (1.7–2.1)
State subsidized care^a^								
No	26,820	26.5 (26.2–26.7)	15,933	15.7 (15.5–15.9)	4506	4.5 (4.3–4.6)	1958	1.9 (1.9–2.0)
Yes	4700	23.6 (23.0–24.2)	3327	16.7 (16.2–17.2)	973	4.9 (4.6–5.2)	428	2.2 (2.0–2.4)

Abbreviations: BP, blood pressure; CI, confidence interval.

a Beneficiary of Medi-Cal or other state subsidized support programs.

**Figure 2:**
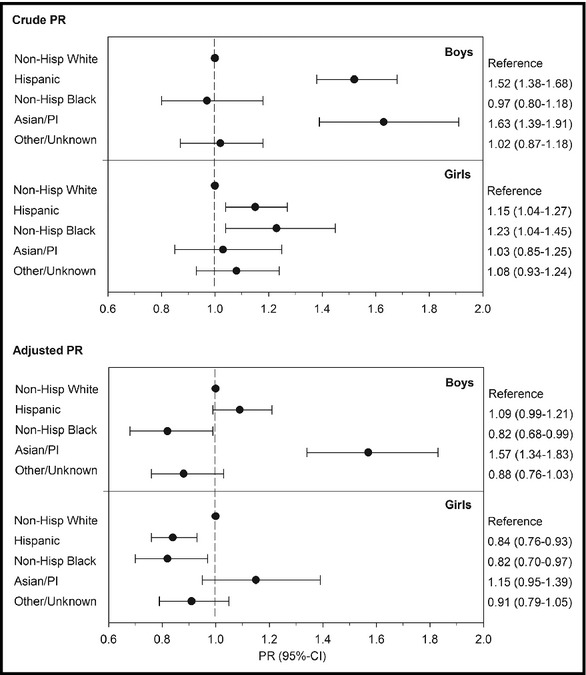
Crude and adjusted prevalence ratio (PR) for hypertension in youth aged 6 to 17 years by race and sex (*P* for interaction sex × race=.001) suggesting that racial disparities in hypertension are stronger in boys than in girls and are significantly attenuated after adjustment for other factors including body mass index for age. PI indicates Pacific Islander; CI, confidence interval.

The PR for hypertension varied by race/ethnicity (Table 4). The crude PR of hypertension was higher in Hispanic, black, and Asian youth than in their non-Hispanic white counterparts. The racial disparities in hypertension risk were substantially attenuated for Hispanic and black youth after adjustment for age, sex, and body weight. The racial disparities in hypertension risk were stronger among boys than among girls (*P* for interaction sex × race=.001, Figure 2).

**Table IV:** Hypertension Among Youths Aged 6 to 17 Years in Southern California by Defined Factors

	BP in the Hypertensive Range		Hypertension	
	Crude PR (95% CI)	Adjusted PR (95% CI)	Crude PR (95% CI)	Adjusted^a^ PR (95% CI)
Total	–	–	–	–
Sex				
Male	1.00 (reference)	1.00 (reference)	1.00 (reference)	1.00 (reference)
Female	0.90 (0.89–0.92)	0.98 (0.96–0.99)	0.86 (0.81–0.91)	1.02 (0.97–1.08)
Age group, y				
6–11	1.22 (1.20–1.24)	1.15 (1.14–1.17)	1.03 (0.98–1.09)	0.96 (0.91–1.01)
12–17	1.00 (reference)	1.00 (reference)	1.00 (reference)	1.00 (reference)
Race/ethnicity				
Non-Hispanic white	1.00 (reference)	1.00 (reference)	1.00 (reference)	1.00 (reference)
Hispanic	1.12 (1.10–1.14)	0.97 (0.95–0.98)	1.33 (1.24–1.43)	0.96 (0.90–1.03)
Black	1.09 (1.06–1.13)	0.96 (0.93–0.99)	1.11 (0.98–1.25)	0.83 (0.73–0.94)
Asian or Pacific Islander	1.10 (1.07–1.14)	1.09 (1.06–1.13)	1.34 (1.19–1.52)	1.36 (1.21–1.53)
Other/unknown	1.06 (1.03–1.09)	1.00 (0.97–1.02)	1.05 (0.95–1.16)	0.90 (0.81–1.00)
Age 6–11 y	–	–	–	–
Sex				
Male	1.00 (reference)	1.00 (reference)	1.00 (reference)	1.00 (reference)
Female	1.01 (0.99–1.03)	1.06 (1.04–1.09)	1.02 (0.94–1.11)	1.18 (1.09–1.29)
Race/ethnicity				
Non-Hispanic white	1.00 (reference)	1.00 (reference)	1.00 (reference)	1.00 (reference)
Hispanic	1.07 (1.04–1.11)	0.95 (0.93–0.98)	1.32 (1.18–1.48)	0.97 (0.86–1.09)
Black	1.02 (0.97–1.07)	0.93 (0.89–0.98)	1.02 (0.83–1.25)	0.80 (0.65–0.98)
Asian or Pacific Islander	1.09 (1.04–1.15)	1.09 (1.04–1.14)	1.51 (1.27–1.80)	1.49 (1.25–1.78)
Other/unknown	1.05 (1.00–1.09)	0.99 (0.96–1.04)	1.01 (0.85–1.20)	0.88 (0.74–1.04)
Age 12–17 y	–	–	–	–
Sex				
Male	1.00 (reference)	1.00 (reference)	1.00 (reference)	1.00 (reference)
Female	0.84 (0.82–0.85)	0.91 (0.89–0.93)	0.76 (0.71–0.82)	0.92 (0.85–0.98)
Race/ethnicity				
Non-Hispanic white	1.00 (reference)	1.00 (reference)	1.00 (reference)	1.00 (reference)
Hispanic	1.12 (1.09–1.15)	0.97 (0.95–1.00)	1.33 (1.22–1.46)	0.95 (0.87–1.04)
Black	1.12 (1.07–1.18)	0.98 (0.94–1.02)	1.17 (1.00–1.37)	0.85 (0.72–0.99)
Asian or Pacific Islander	1.06 (1.01–1.11)	1.09 (1.04–1.14)	1.20 (1.01–1.41)	1.25 (1.06–1.47)
Other/unknown	1.06 (1.02–1.10)	0.99 (0.96–1.03)	1.07 (0.94–1.22)	0.92 (0.81–1.04)

Abbreviations: BP, blood pressure; CI, confidence interval.

a All prevalence ratios (PRs) are adjusted for sex, age, race/ethnicity, state subsidized care, and body weight class.

## Discussion

In this study conducted in an integrated health care system, 2.1% of youth had hypertension and 31.4% had prehypertension based on routinely collected clinical data. A significant proportion of youth (21.4%) had BP in the hypertensive range; 4.8% nearly met the definition of hypertension with 2 documented hypertensive BP measurements. The present population-based cross-sectional study provides valuable insight into the prevalence of prehypertension and hypertension in youth based on repeated BP measurements in clinical settings where mostly automated BP devices are used.

The prevalence of hypertension estimated from routine BP screening in this study based on 4 consecutive BPs in a clinical outpatient setting is lower than estimated from nonclinical studies^13^,^14^ but higher than estimated from another clinical study.^16^ Nationwide estimates from the National Health and Nutrition Examination Survey (NHANES III) 1988–1994 and NHANES 1999–2002 indicate that 3.7% of youth aged 8 to 17 years have a BP in the hypertensive range and 10.0% have prehypertension.^13^–^14^ However, in that study, BP was measured at only one occasion and the prevalence of hypertension cannot be determined among those with BP in the hypertensive range. In a study conducted in a Texas school-based setting, 15.7% of youth aged 11 to 17 years had prehypertension and 3.2% had hypertension.^17^ However, in that study, the definition of prehypertension included youth with 1 or 2 BPs in the hypertensive range but no hypertension. In a population-based study using clinical data from pediatric practices, 12.7% had prehypertension, 5.3% had BP(s) in the hypertensive range, and 0.1% had hypertension.^16^ In that study,^16^ the prevalence of hypertension was low because a large proportion of children lacked sufficient follow-up visits to confirm the initial elevated BP. In another clinical setting, the prevalence of hypertension was estimated to be 3.6%, but the timeframe for the follow-up in that study was 7 years, potentially leading to an overestimation of the prevalence of hypertension.^11^ Several differences between epidemiologic studies and clinical data have to be noted. In contrast to epidemiologic studies, BP assessments in a managed care setting, such as in the present study, will be conducted at every visit independent of previous BP results. Considering anxiety and certain health conditions during medical visits, the prevalence of prehypertension as well as the prevalence of 1 or 2 BPs in the hypertensive range assessed as part of routine health care may be higher than in epidemiologic studies. On the other hand, hypertension might be underestimated in clinical data if patients are not followed up on as recommended by NHBPEP^20^ to repeat and confirm a hypertensive BP on at least 2 additional occasions.

The high proportion of youth with 1 or 2 hypertensive BPs >95th percentile is particularly noteworthy. BP is variable in youth.^32^ Many youth may reverse to normal BPs by follow-up visits. The high number of youth with BPs in the hypertensive range may be explained by white-coat hypertension and high BP variability. Future studies are necessary to identify predictors of hypertension that persists in youth.

As for many health conditions, health disparities in pediatric hypertension are a concern. Our data suggest that high BP is more prevalent in Hispanic and Asian youth. However, racial disparities were mainly driven by racial differences in obesity rates and were substantially attenuated after adjustment for body weight. These results are consistent with results reported from a school-based screening study.^33^ The higher risk for Asians/Pacific Islanders observed in our study remained significant even after adjustment for body weight. A recent study with a comparable study setting also reported a higher risk of hypertension in Asian/Pacific Islander youth but not in blacks.^16^ These observations are in contrast to adult hypertension where the risk is highest among blacks.^34^,^35^

## Study Limitations

There are several potential differences to other studies that should be noted. As in many clinical settings, BP in our study was primarily measured by automated devices based on an oscillometric technique. In a few cases and for repeated readings, auscultatory devices may have been used but the device type is not recorded in the EHR. We can also not exclude that deviations from the recommended method to assess BP have occurred. Current reference values for BP are based on auscultatory methods.^37^ Studies with older instruments suggest that oscillometric devices may overestimate BP in children, which may lead to an overestimation of youth with prehypertension and hypertension.^38^,^39^ In adults, oscillometric devices tend to slightly underestimate BP.^41^ On the other hand, manual auscultatory measurement of BP during clinical routine also leads to a significant error as a result of measurement inaccuracies in the rush of daily routine, terminal digit preference, and noise in the examination room that may influence the ability to accurately estimate the onset of Korotkoff sounds.^42^

Because no home monitoring of BP was conducted, we were not able to assess masked hypertension, which may be present in about 3% to 4% of youth^43^ and has been shown to be associated with structural cardiac changes.^44^ White-coat hypertension is characterized by a persistently elevated office BP and a persistently normal daytime ambulatory BP, found in up to 12% of children and usually not associated with end-organ damage.^45^ The presence of white-coat hypertension may have led to an overestimation of the prevalence of high BP in our study. Our cohort was slightly older than the source population because of the exclusion of children who did not have at least 3 BPs measurements from separate visits. However, this age difference will not affect age-specific estimates. Moreover, these differences based on exclusion criteria were not sufficient to account for the differences in our estimates of the prevalence of prehypertension and hypertension compared with other studies.

Another potential limitation lies in the fact that we did not exclude medical visits related to any health conditions that may lead to a slightly higher BP. Nor did we limit BPs to those measured in healthy child visits, as has been done by others.^11^ However, we limited our data to those from nonurgent outpatient visits and excluded BPs from medical visits during which there was an indication of the presence of fever, which is known to be associated with greater BP.^46^ The rationale for the decision to include all nonurgent outpatient visits except those indicating fever—in contrast to healthy child visits only—was that healthy child/adolescent visits are rare in older youth, and limiting the cohort to youth with at least 3 well-child visits would, therefore, lead to a substantial underestimation of the prevalence of high BP in adolescents. On the other hand, we cannot exclude that some BPs were elevated secondary to acute conditions or undiagnosed chronic conditions. This, however, is unlikely to have affected the estimates of the prevalence of hypertension because this classification requires 2 confirmatory elevated BPs measured in subsequent visits. However, this may have resulted in an overestimate of the prevalence of prehypertension, especially of prehypertension at a single occasion.

## Study Strengths

The present study benefited from routine BP assessments conducted during medical office visits as opposed to single or a defined number of repeated measurements in epidemiologic studies. Standardized protocols and regular training of staff were implemented, although deviations from the standardized protocol cannot be excluded. All children were members of a large prepaid managed care system and standardized screening guidelines for BP in all children in the health plan were routine. As a result of multiple measurements over a longer period, we were able to evaluate the prevalence of hypertension based on the first 4 BPs, and to provide estimates of the proportion of youth who continued to have elevated BP on subsequent visits.

To recognize and address pediatric hypertension is important because the consequences of pediatric hypertension can occur in childhood or early adulthood,^8^–^9^ which can be especially severe when hypertension is left untreated. There are several reports of early end-organ damage in children with hypertension. These include increased intima-media thickness,^9^ left ventricular hypertrophy,^7^,^9^ arterial stiffness,^9^–^48^ and other structural cardiac changes.^49^ Some structural cardiac changes as a result of high BP have been noted as early as 2 years of age.^49^ In adults, hypertension is a major risk factor for cardiovascular disease, stroke, and kidney damage.^20^ Studies also suggest that youth with hypertension have neurocognitive alterations manifesting as learning difficulties and cognitive dysfunction.^50^–^51^ Thus, better knowledge of the prevalence of hypertension, including the earlier stages of prehypertension during childhood, can help to quantify the magnitude of the problem and identify those subgroups that are at the highest risk for hypertension. Further studies are necessary to understand the health risks associated with prehypertension and hypertension and BP variability in youth.

## Conclusions

The present study suggests that an alarming number of youth have hypertension or nearly meet the definition of hypertension with 2 documented BPs in the hypertensive range. This may prove important for the stratification of cardiovascular risk and in the development of optimal screening strategies for high BP in children. Screening youth at the highest risk to progress to persistent hypertension, and the early initiation of interventions, could decrease the rate of progression of hypertension and reduce cardiovascular consequences of hypertension in adulthood.
